# National Medical Association

**Published:** 1860-11

**Authors:** 


### EDITORIAL AND MISCELLANEOUS.

### The National Medical Association.

Under this head, the accomplished Editor of the Medical Journal of North Carolina, Dr. Edward Warren, deals some heavy blows upon the wire-workers of the last session of the American Medical Association at New Haven. We insert the entire article, and would call especial attention to the last paragraph, which alludes to the only plan, in our opinion, by which the profession can be protected from those not qualified to enter the ranks. He says:

"This body met in New Haven on the 5th of June last, and was well attended by physicians from all sections of the country. As usual, most of the time was expended in the discussion of matters which had no special relation to Medicine—though they seemed real "God-sends" to some of the members, inasmuch as they afforded them an opportunity for the indulgence of that loquacity which seems to be the special failing of many of our medical men.

The subject of Medical Education received a thorough ventilation, and the old contest between the *ins* and the *outs* was renewed with redoubled vigor. We are as warm an advocate for "reform" as any man, but we are not prepared to pander to the insane prejudice which many persons have manifested in this regard—especially when we believe it has originated solely in selfish and sordid motives. The Report of the Committee on Education—which was read and prepared by Dr. Reese, of New York—was characterized by that ability and independence which distinguish all that emanates from his pen. It contains many striking facts and valuable suggestions, and was in every respect, worthy of the subject and of its distinguished author. We voted for its publication with pleasure, and will lay it before our readers when it makes its appearance in print; but we must say that some of the resolutions which received the sanction of the Convention met with our most decided disapprobation, and that, though we stood solitary and alone, our vote was recorded against them. We were not willing to place the Colleges of the country, whatever may be their defects, at the mercy of the selfish and envious cliques which not unfrequently surround them. We could not consent to have the University of Virginia, one of the best schools in this or any other country, deprived of the right of representation in the National Medical Association. We could not sanction a movement which, from its very stringency and harshness, must prove entirely inoperative—"keeping the word of promise to the ear, and breaking it to the hope," of the Profession; and we felt bound, by every obligation of honor and of duty, to oppose the resolutions at the time—even though they were the special pets of the Association—and to expose their fallacies now, however it may prove to their special friends and champions.

Bad legislation is worse than no legislation at all, and we feel assured that the attempts at improvement in Medical Education, recently made by this august body, so far from accomplishing the ends at which they are aimed, will only serve to retard and embarrass the reforms which all acknowledge to be most necessary and desirable.

The best way to insure the entrance of proper men into the ranks of our Profession consists in subjecting those who desire that honor, to a thorough, satisfactory and impartial examination; and we feel confident that North Carolina has done more towards the accomplishment of this result, by the establishment of her Board of Independent Medical Examiners, than the Medical Association will accomplish by a century of such legislation as has distinguished it of late. Let other States imitate our example, and the good work will be finished, long before the wranglers who delight in spreading themselves at each annual meeting of the National Convention have concluded their senseless and unprofitable controversies.

